# Supplementary material for: Electrostatic force promoted intermolecular stacking of polymer donors toward 19.4% efficiency binary organic solar cells
Source: Nat Commun. 2023 Oct 9;14:6297. doi: 10.1038/s41467-023-42071-2 (PMC10562425; doi:10.1038/s41467-023-42071-2)
Supplement: Supplementary file 1 — Supplementary Information [file 41467_2023_42071_MOESM1_ESM.pdf]

Supplementary Information

Electrostatic force promoted intermolecular stacking of polymer donors toward 19.4% efficiency binary organic solar cells

Zirui Gan[1], Liang Wang[1], Jinlong Cai[1], Chuanhang Guo[1], Chen Chen[1], Donghui Li[1], Yiwei Fu[1], Bojun Zhou[1], Yuandong Sun[1], Chenhao Liu[1], Jing Zhou[1], Dan Liu[1], Wei Li[1], Tao Wang[1,2*]

[1]School of Materials Science and Engineering, Wuhan University of Technology, Wuhan 430070, China

[2]School of Materials and Microelectronics, Wuhan University of Technology, Wuhan 430070, China

E-mail: twang@whut.edu.cn

Supplementary Methods

Synthesis of INMB-F

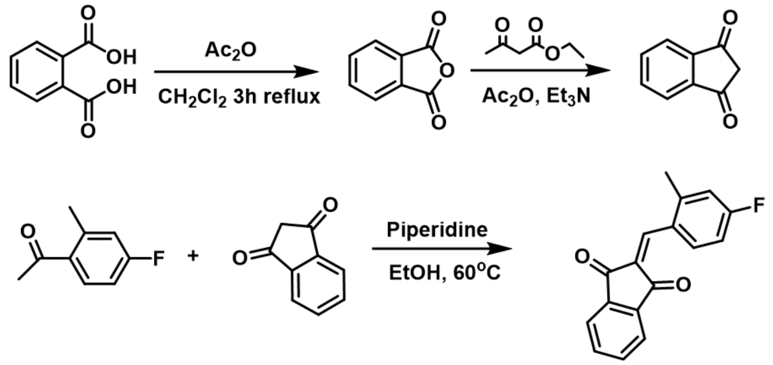

Supplementary Fig. 1. Synthesis routes of conjugated molecule INMB-F.

The characterization of these intermediates was reported in previous work as well [1]. To a mixed solution of 1-(4-fluoro-2-methylphenyl)ethan-1-one (1.24 g, 10 mmol),1H-indene-1, 3(2H)-dione (1.46 g, 10mmol) in absolute ethyl alcohol (50 ml) was added piperidine (85.15 mg, 1 mmol). The mixture was allowed to stir and reflux at 60 °C for 3 h. After cooling down to room temperature, the precipitate was filtered and washed with EtOH. The residue was further purified using column chromatography on silica gel employing CH_2Cl_2 as an eluent to afford a pure red solid (1.35 g, 55%) ^{1}H NMR (500 MHz, CDCl3) 8.73 (1 H, s), 8.15 (1 H, s), 8.04 - 7.96 (2 H, m), 7.82 (2 H, d, J 8.6), 7.00 (2 H, d, J 14.6), 2.56 (3 H, s). ^{13}C NMR (126 MHz, CDCl3) δ 190.17, 188.92, 166.23, 164.19, 144.56, 144.49, 142.74, 142.46, 140.01, 135.44, 135.28, 128.69, 127.81, 123.35, 123.33, 117.62, 117.45, 113.37, 113.20, 20.54. ^{19}F NMR (471 MHz, CDCl3) δ -104.96 -105.05 (m).

Supplementary Figures

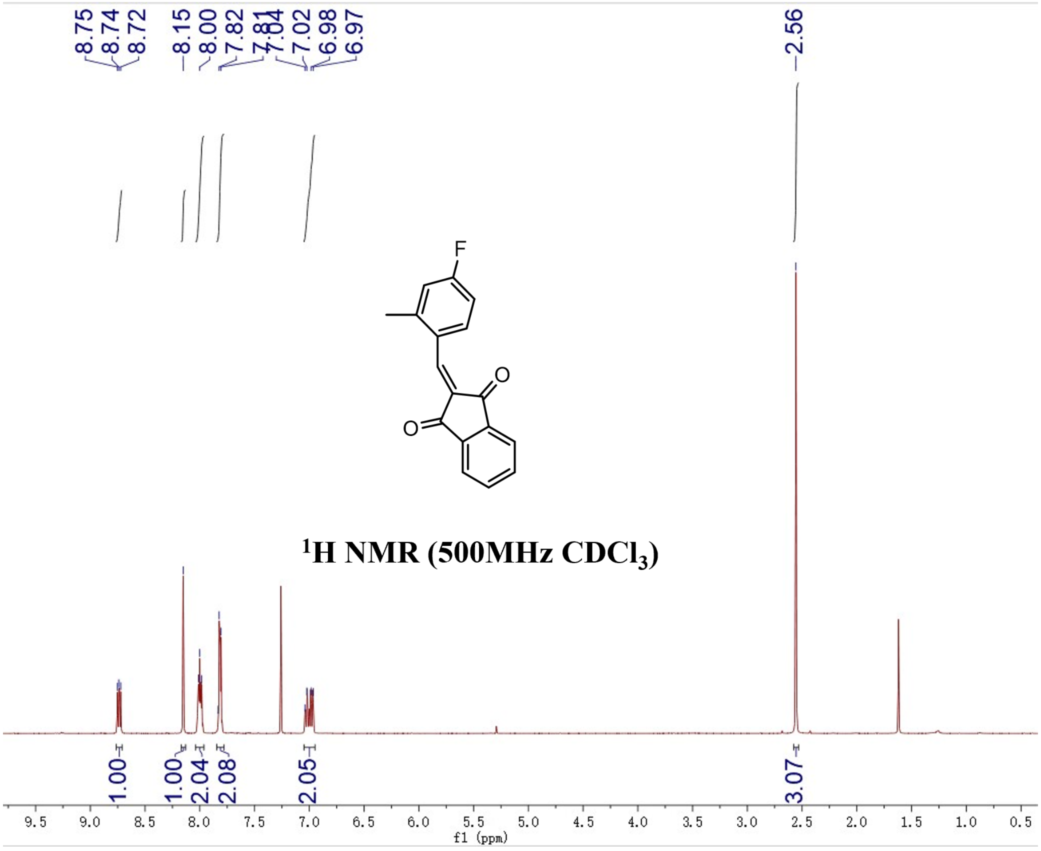

Supplementary Fig. 2. ^{1}H NMR of INMB-F.

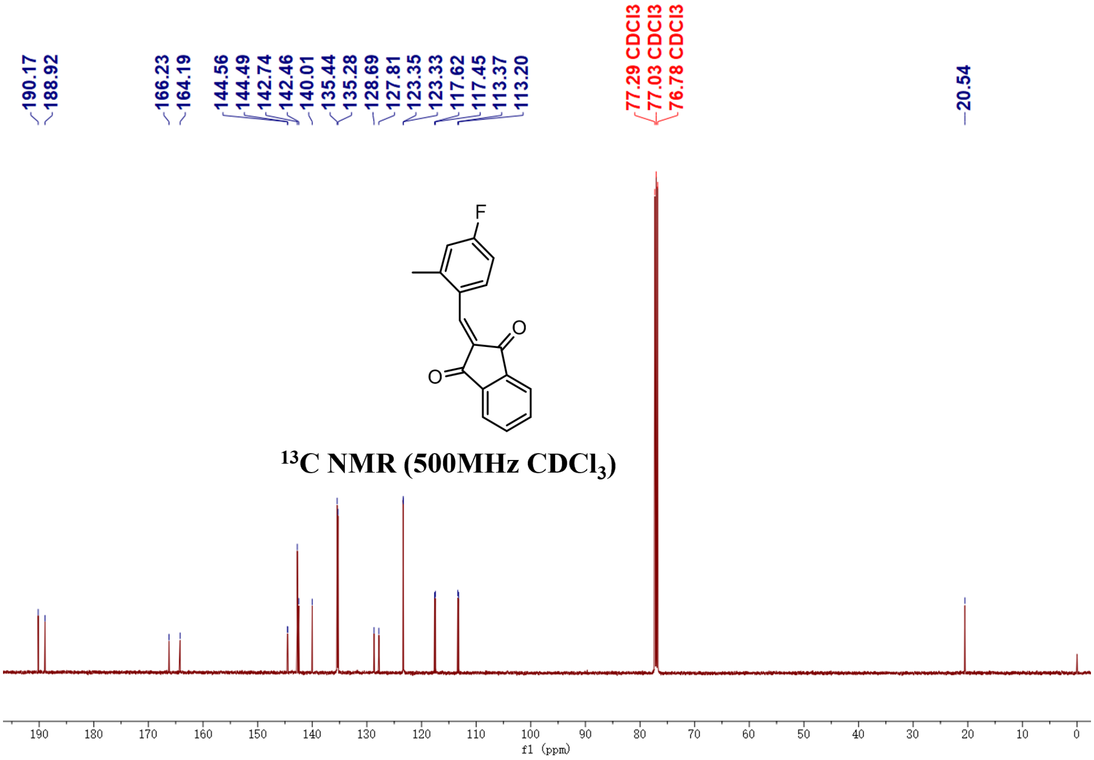

Supplementary Fig. 3. ^{13}C NMR of INMB-F.

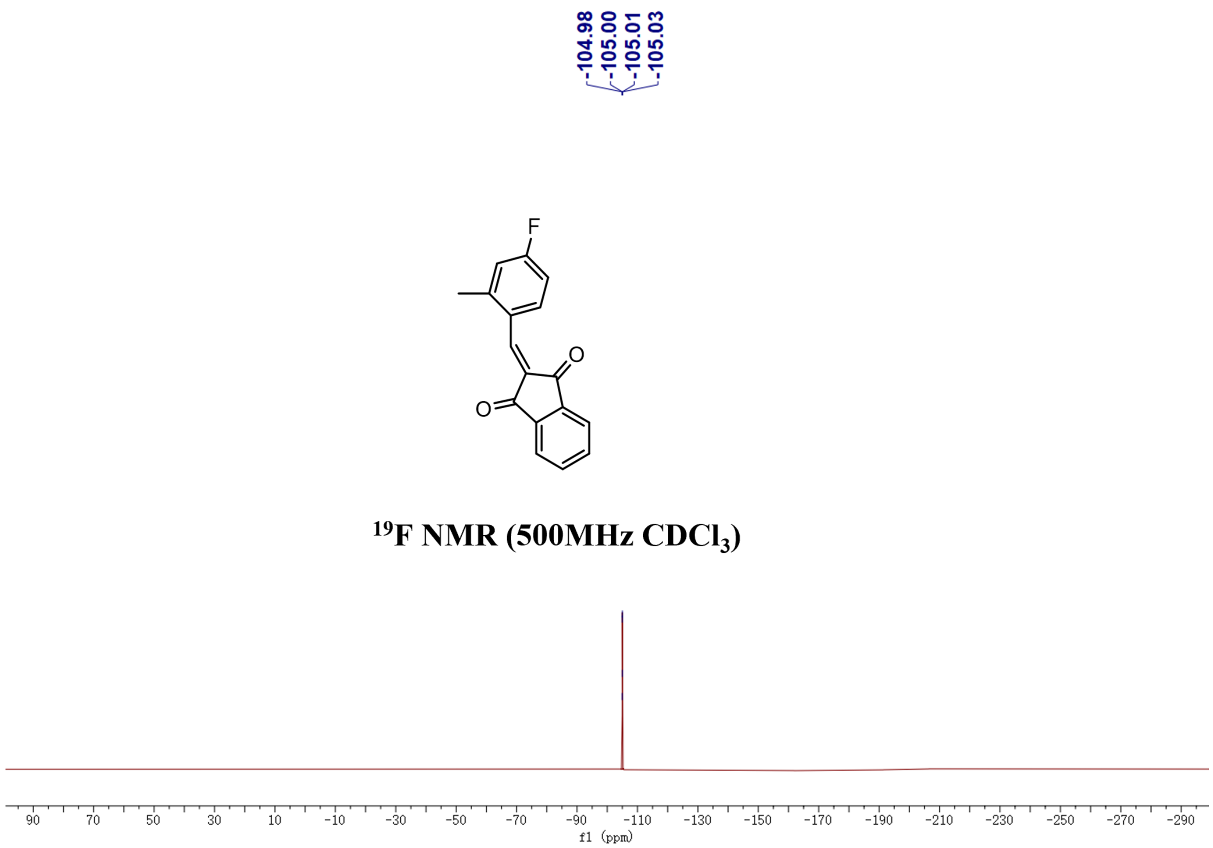

Supplementary Fig. 4. ^{19}F NMR of INMB-F.

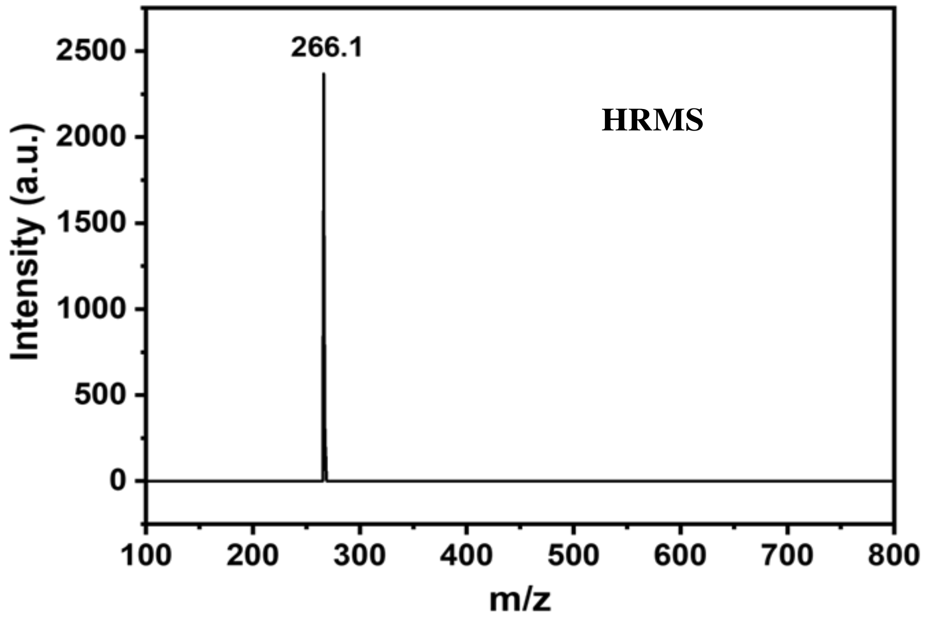

Supplementary Fig. 5. High-resolution mass spectrum of INMB-F.

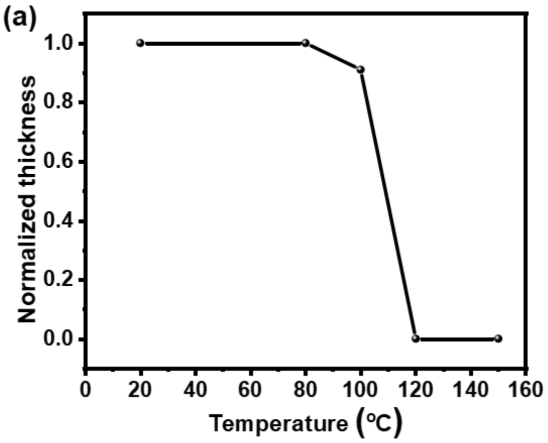

Supplementary Fig. 6. (a) Thickness of pure INMB-F films after heating at different temperatures for 10 min and (b) Thickness of pure INMB-F films upon heating at 120 °C for different time.

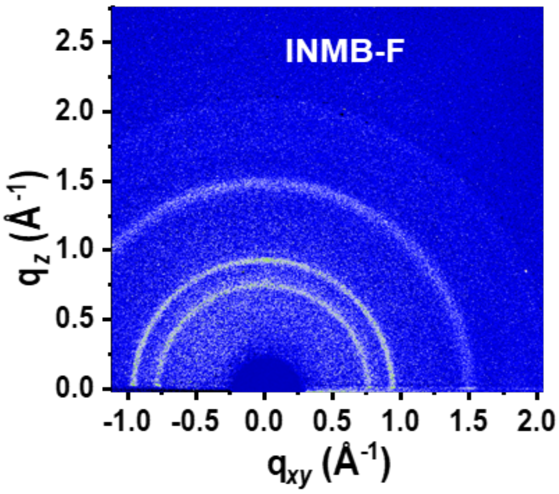

Supplementary Fig. 7. 2D GIWAXS pattern of INMB-F.

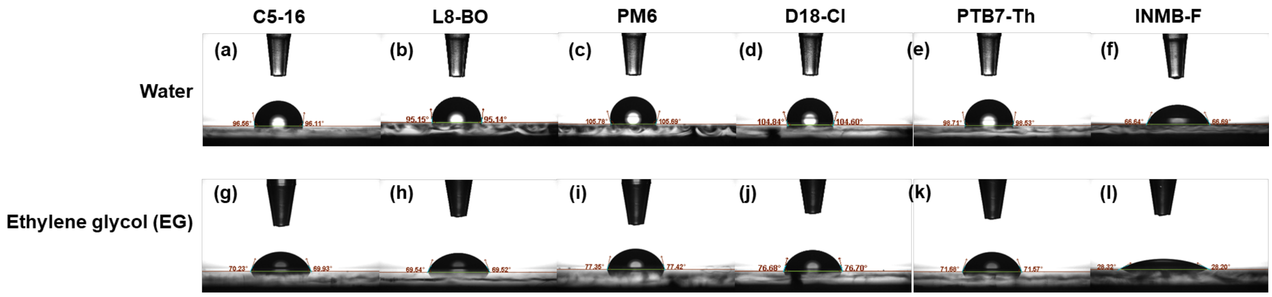

Supplementary Fig. 8. Contact angle images of (a, g) C5-16, (b, h) L8-BO, (c, i) PM6, (d, j) D18-Cl, (e, k) PTB7-Th and (f, l) INMB-F neat films with water or ethylene glycol droplet on top.

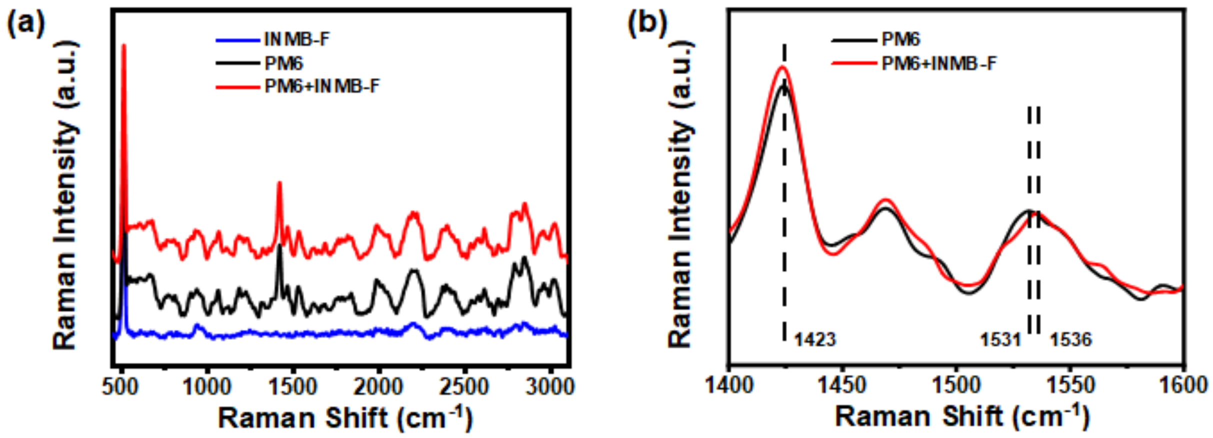

Supplementary Fig. 9. Raman spectra of pure INMB-F film, PM6 film with and without the addition of INMB-F, (a) in the range of 450-3200 cm^{-1}. (b) in the range of 1400-1600 cm^{-1}.

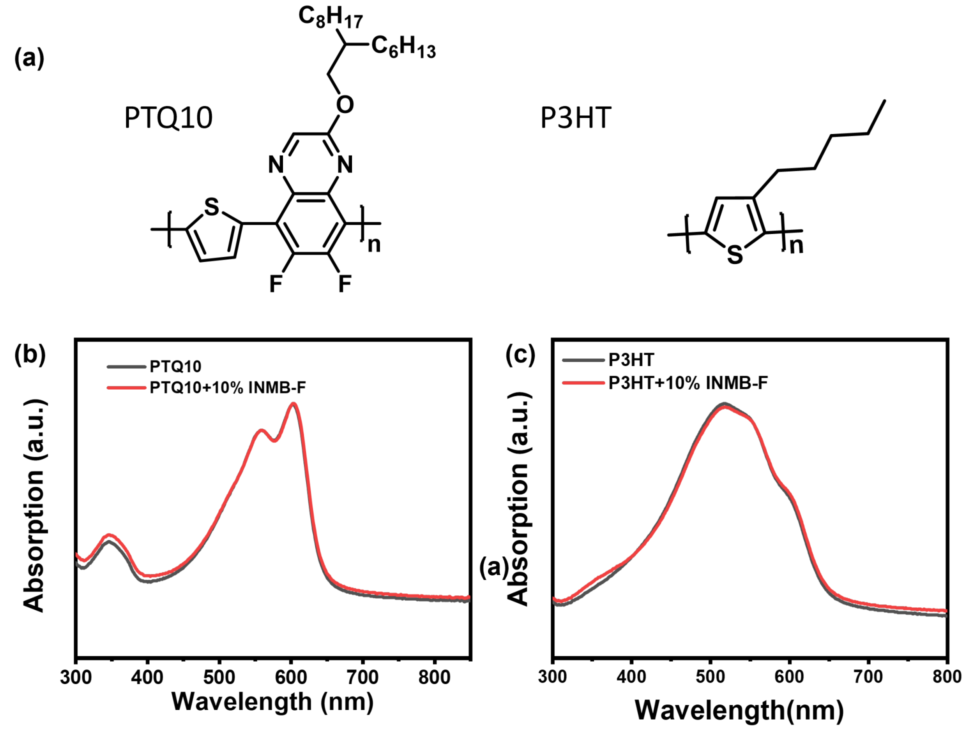

Supplementary Fig. 10. (a) Molecular structure of PTQ-10 and P3HT. Absorption spectra of (b) PTQ-10 and (c) P3HT with the addition of INMB-F.

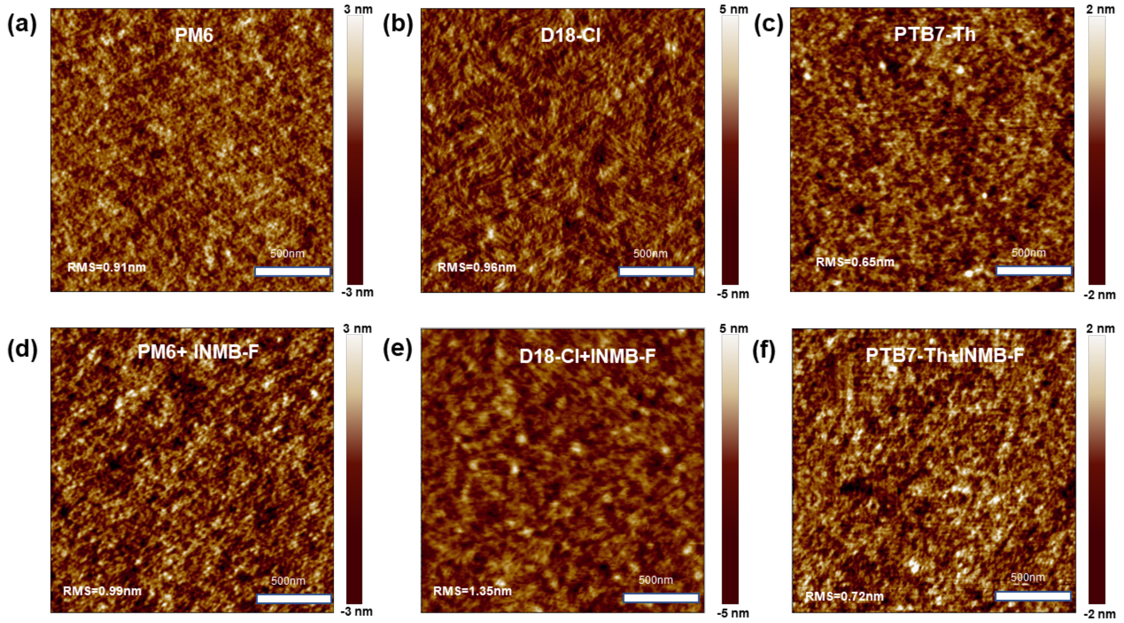

Supplementary Fig. 11. AFM images of (a, d) PM6 with and without INMB-F, (b, e) D18-Cl with and without INMB-F, and (c, f) PTB7-Th with and without INMB-F.

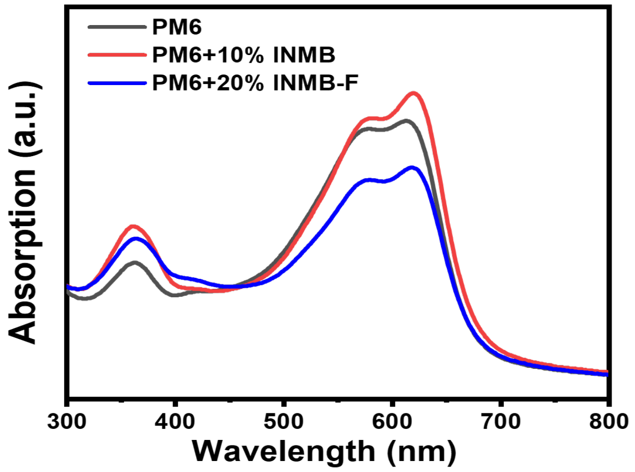

Supplementary Fig. 12. UV-Vis absorption of PM6 film with different amount INMB-F.

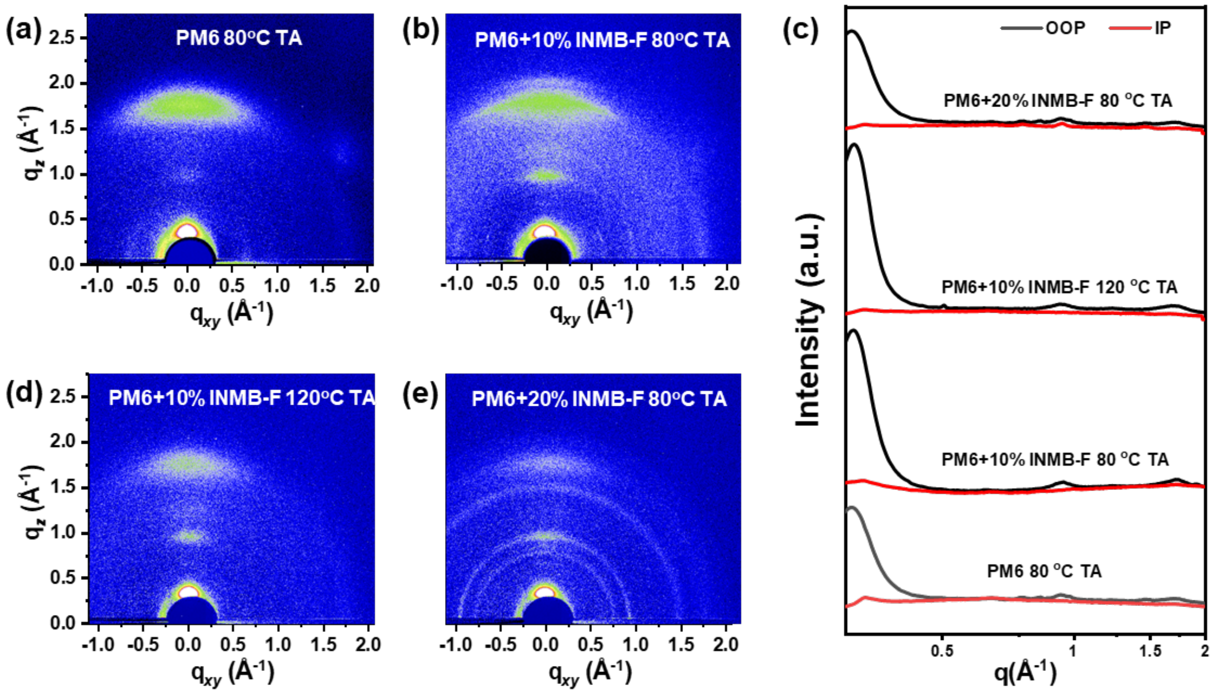

Supplementary Fig. 13. (a, b, d, e) 2D GIWAXS patterns and (c) 1D profiles of PM6 films with different amount of INMB-F upon different TA temperatures.

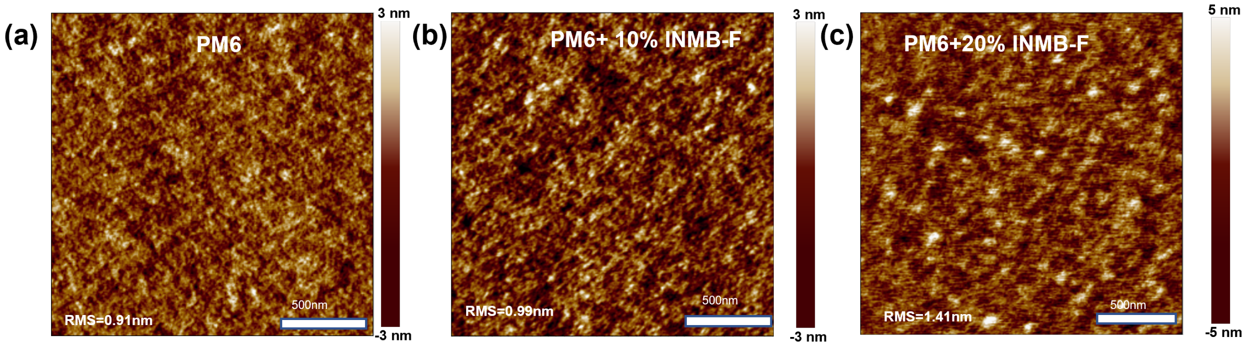

Supplementary Fig. 14. AFM images of PM6 films (a) without and with (b) 10% or (c) 20 % of INMB-F.

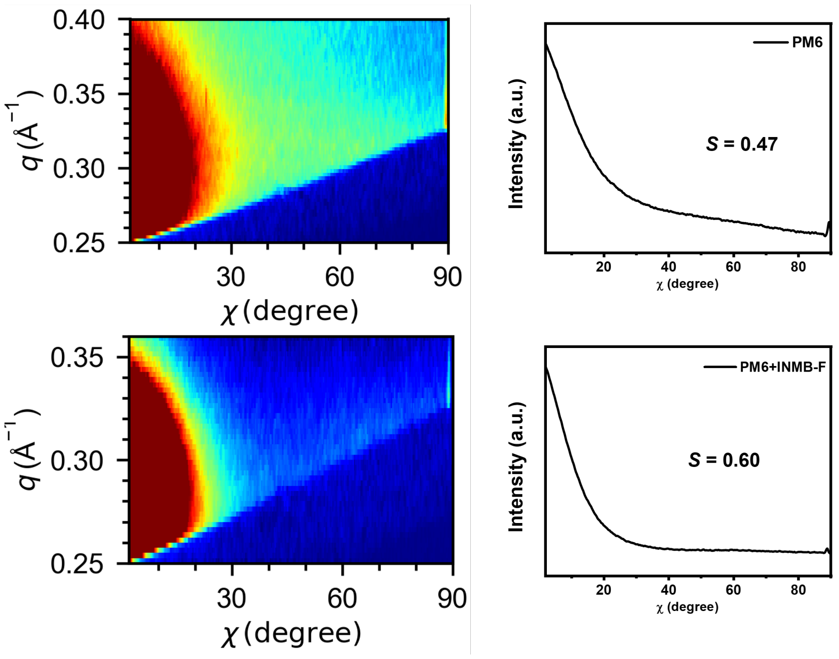

Supplementary Fig. 15. Quasi-pole figure and image plots of the (100) lattice plane of PM6 without and with INMB-F.

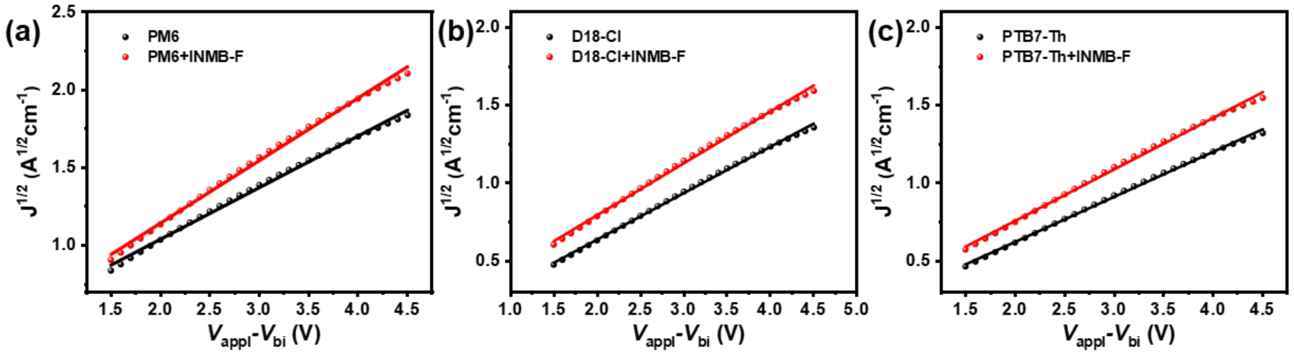

Supplementary Fig. 16. Hole mobilities of (a) PM6 with and without INMB-F, (b) D18-Cl with and without INMB-F and (c) PTB7-Th with and without INMB-F.

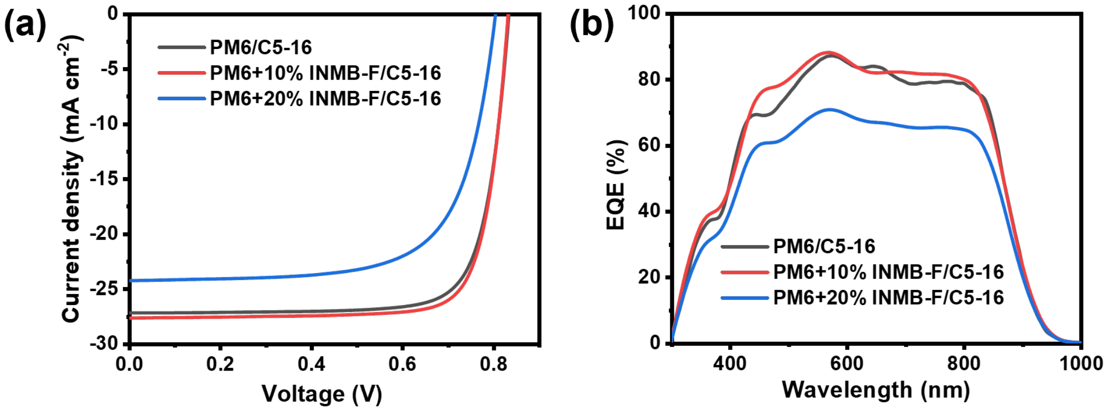

Supplementary Fig. 17. (a) *J-V* curves and (b) EQE spectra of PM6/C5-16 devices with different amounts of INMB-F.

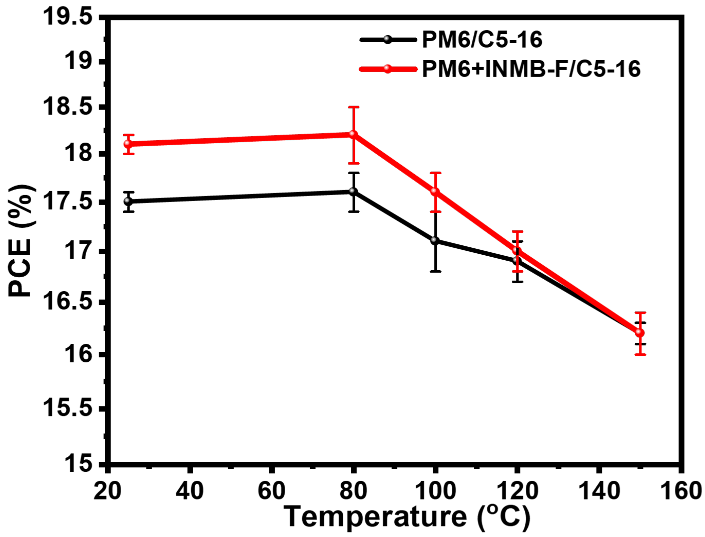

Supplementary Fig. 18. Efficiency variation of PM6/C5-16 OSCs with and without INMB-F under different thermal annealing temperatures.

Test Report

Test Report No. AGXB123W00327

Subject unit	Organic Solar Cells-2
Manufacturer	Wuhan University of Technology
Commission unit	Tao Wang
Inspection category	Commissioned inspection

Chengdu Institute of Product Quality Inspection Co., Ltd.

National Photovoltaic Product Quality Inspection & Testing Center

Chengdu Institute of Product Quality Inspection Co., Ltd.
National Photovoltaic Product Quality Inspection &Testing Center
TEST REPORT

Test Report No.AGXB123W00327

Product Name	Organic solar cells-2	Trade Mark	/
Manufacture Date	14/08/2023	Model /Type	0.0404cm^2
Sample No.	AGXB123W00327	Sample Grade	/
Sample Quantity	One piece	Sample State	/
Delivery Date	15/08/2023	Sample Delivered personnel	Tao Wang
Commission unit	Tao Wang	Manufacturer	Wuhan University of Technology
Commission unit address	School of Materials Science and Engineering, Wuhan University of Technology, No. 122 Luoshi Road, Wuhan 430070, P. R. China	Manufacturer Address	No. 122 Luoshi Road, Wuhan 430070, P. R. China
Commission unit Zip code	430070	Manufacturer Zip code	430070
Commission unit Tel.	18507131726	Manufacturer Tel.	18507131726
Center Address	No. 355, 2nd Tengfei Road, Southwest Airport Economic Development Zone, Chengdu, Sichuan, P. R. China.	Measurement Date	15/08/2023
Methods	IEC 60904-1:2020 Photovoltaic devices-Part 1: Measurement of Photovoltaic Current-Voltage Characteristics.		
Test conclusion	This column blank. (Special chapter for test report) Issue Date: [illegible]/08/[illegible]		
Remarks	The mask area is provided by the Commission unit: 0.0404cm^2.		

Approved by [illegible] **Reviewed by** [illegible] **Measured by** [illegible]

Chengdu Institute of Product Quality Inspection Co., Ltd.
National Photovoltaic Product Quality Inspection &Testing Center
TEST REPORT

Test Results:

No.	Test item(s)	Unit	Results
1	Current-voltage characteristics measurement	---	---
1.1	Open-circuit voltage, V_{OC}	V	0.877
1.2	Short-circuit current, I_{SC}	mA	1.099
1.3	Maximum-power, P_{max}	mW	0.766
1.4	Maximum-power voltage, $V_{p\text{-}max}$	V	0.749
1.5	Maximum-power current, $I_{p\text{-}max}$	mA	1.023
1.6	Fill factor, FF	%	79.50
1.7	Conversion efficiency, η	%	18.96

Current-voltage characteristics

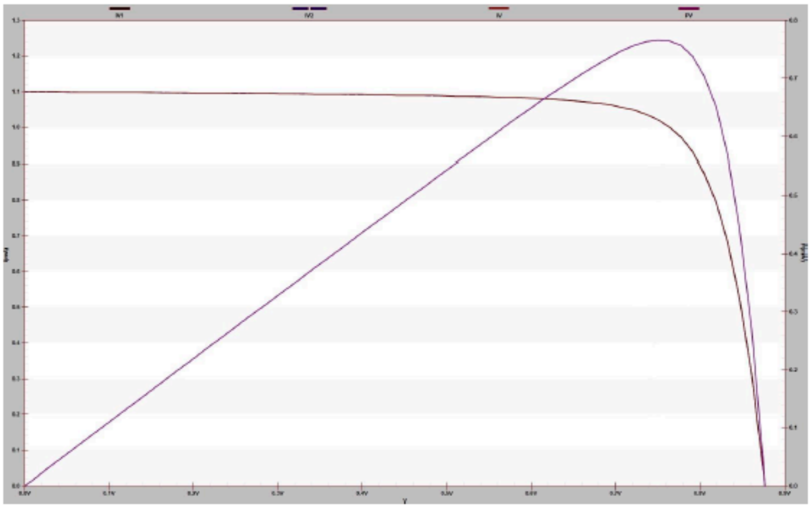

Remark: Sample was tested under the irradiation with a steady-state class calibrated AAA solar simulator (AM1.5-G 1000.0 W/m^2 basted on mono-Si reference cell) at 25±1 °C. Designated area defined by thin metal aperture mask.

——————**Blank**——————

Supplementary Fig. 19. Certification by the National Photovoltaic Product Quality Inspection & Testing Center (China). The device was measured with a mask of 4.04 mm^2 and gave a PCE of 18.96%.

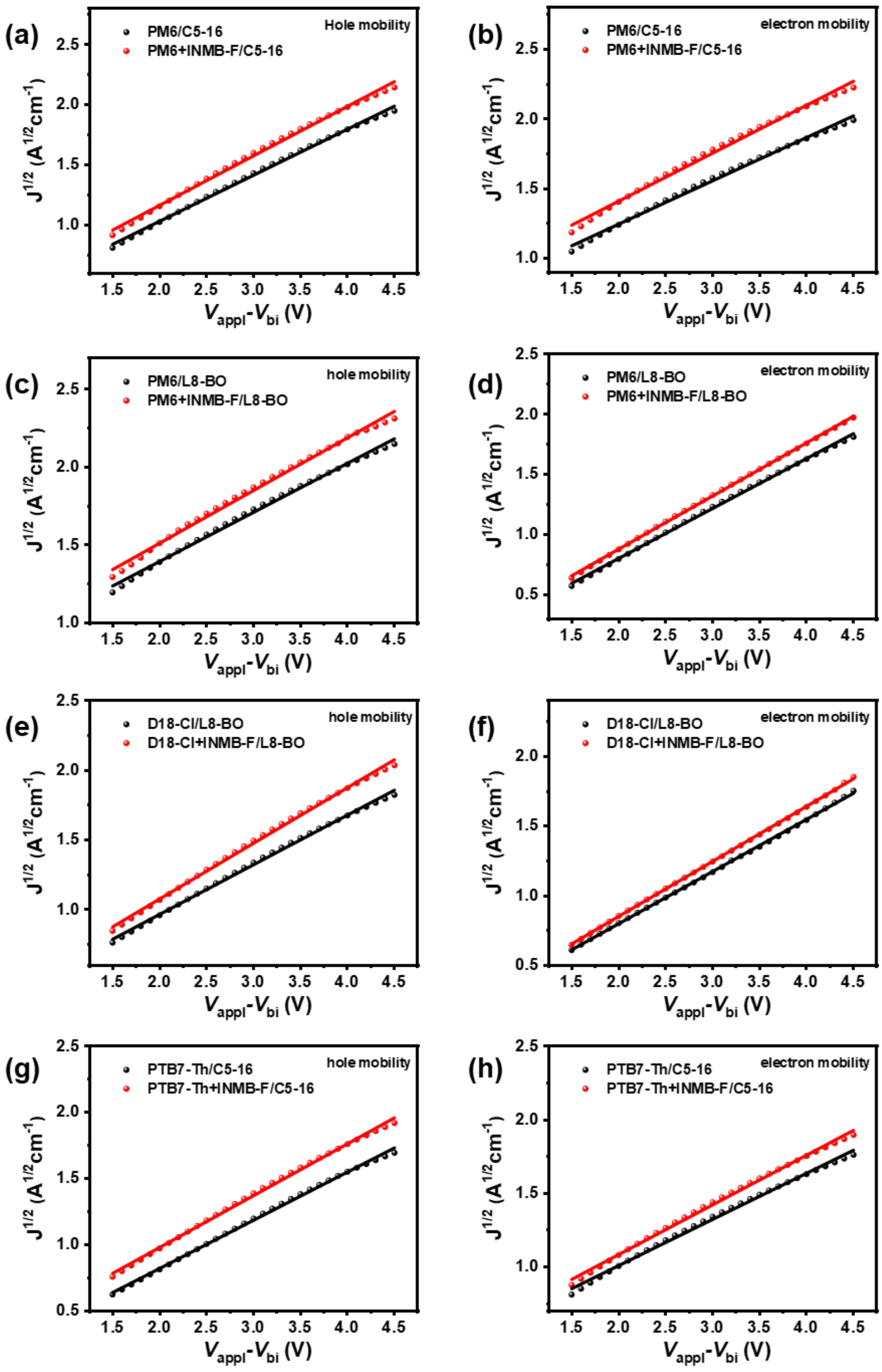

Supplementary Fig. 20. Hole and electron mobilities of (a-b) PM6/C5-16 with and without INMB-F, (c-d) PM6/L8-BO with and without INMB-F, (e-f) D18-Cl/L8-BO with and without INMB-F and (g-h) PTB7-Th/C5-16 with and without INMB-F.

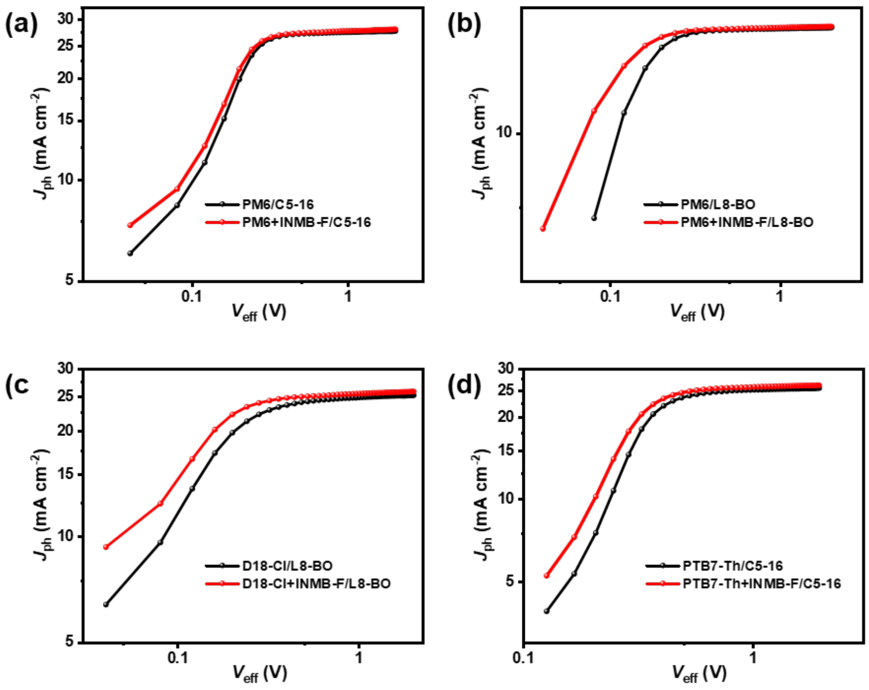

Supplementary Fig. 21. Photocurrent density (J_{ph}) versus effective voltage (V_{eff}) curves of (a) PM6/C5-16 with and without INMB-F, (b) PM6/L8-BO with and without INMB-F, (c) D18-Cl/L8-BO with and without INMB-F and (d) PTB7-Th/C5-16 with and without INMB-F.

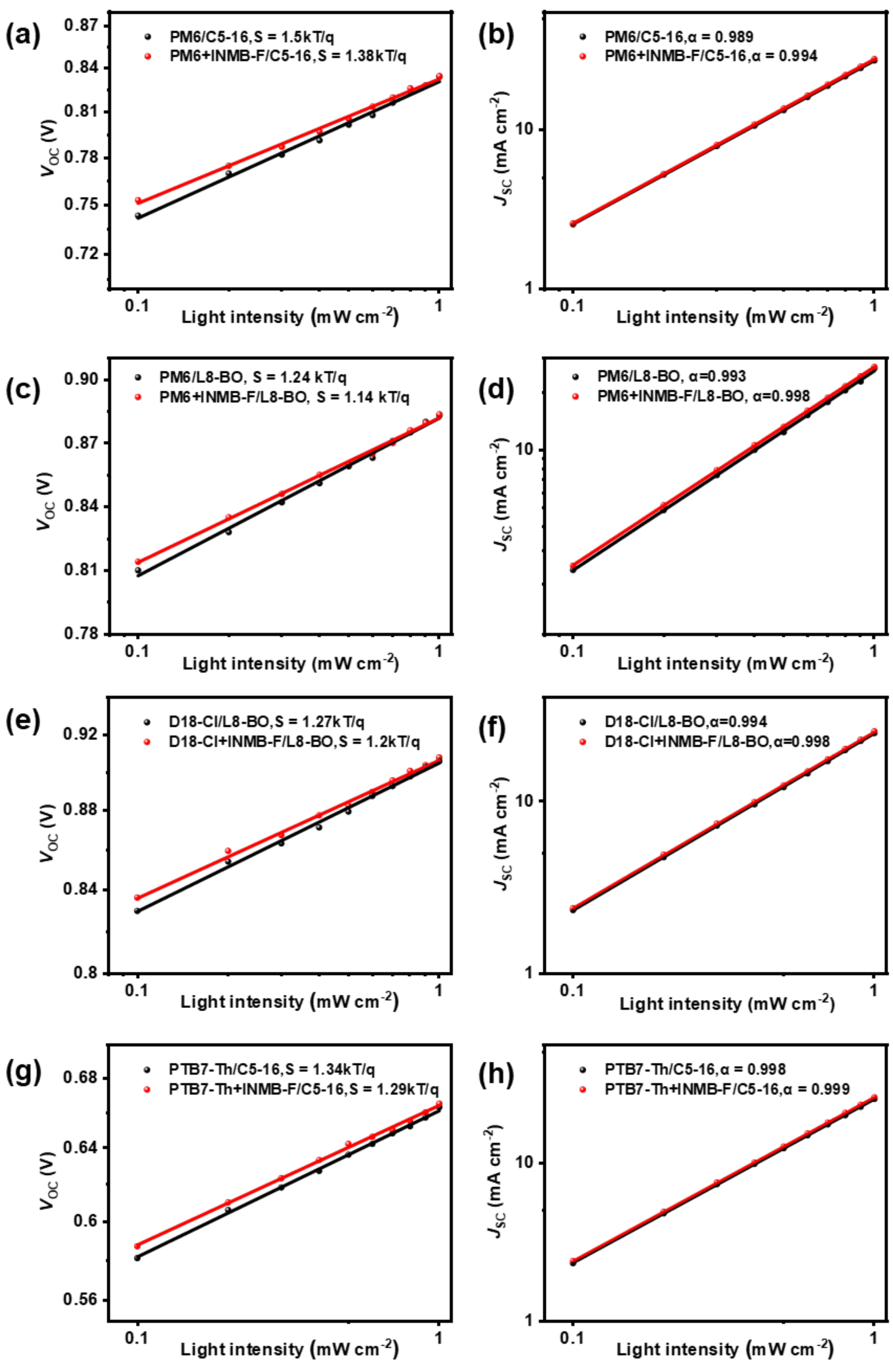

Supplementary Fig. 22. V_{OC} and J_{SC} vs light intensity of (a-b) PM6/C5-16 with and without INMB-F, (c-d) PM6/L8-BO with and without INMB-F, (e-f) D18-Cl/L8-BO with and without INMB-F and (g-h) PTB7-Th/C5-16 with and without INMB-F.

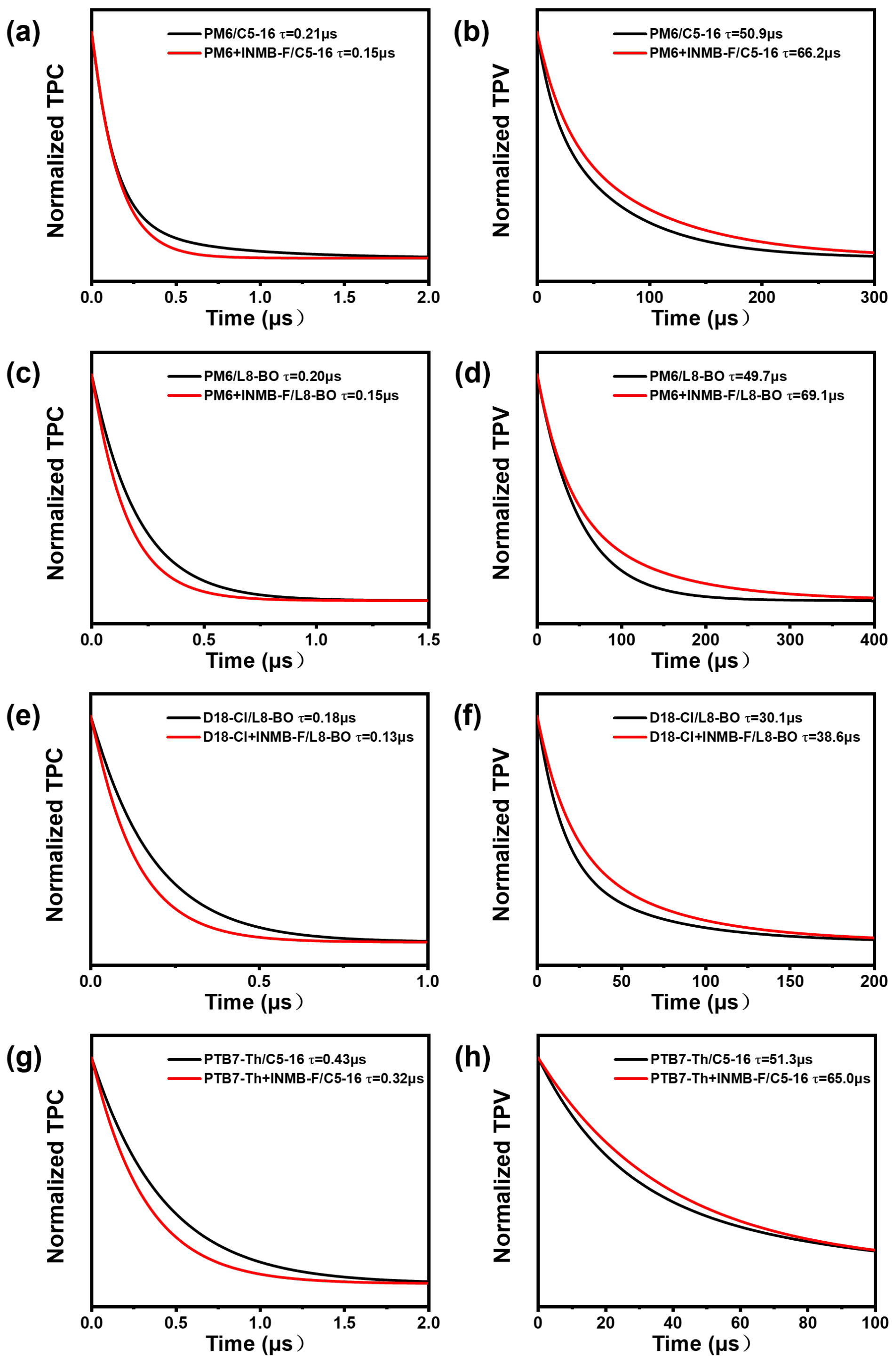

Supplementary Fig. 23. Normalized TPC and TPV of (a-b) PM6/C5-16 with and without INMB-F, (c-d) PM6/L8-BO with and without INMB-F, (e-f) D18-Cl/L8-BO with and without INMB-F and (g-h) PTB7-Th/C5-16 with and without INMB-F.

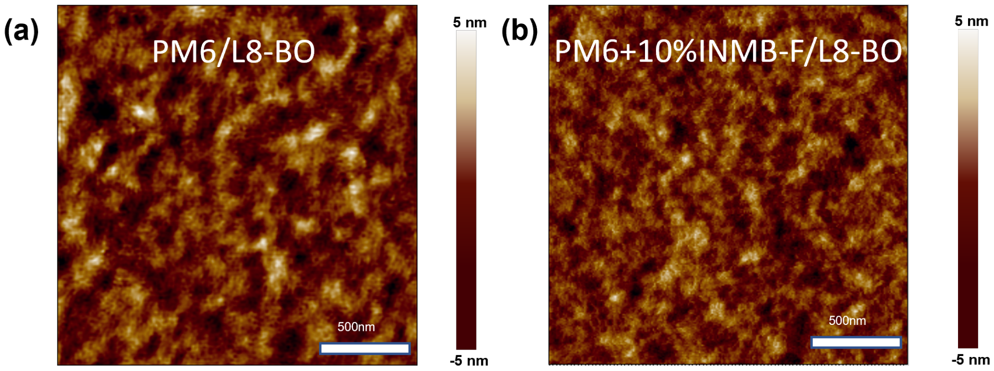

Supplementary Fig. 24. AFM images of (a) PM6/L8-BO and (b) PM6+10% INMB-F/L8-BO films.

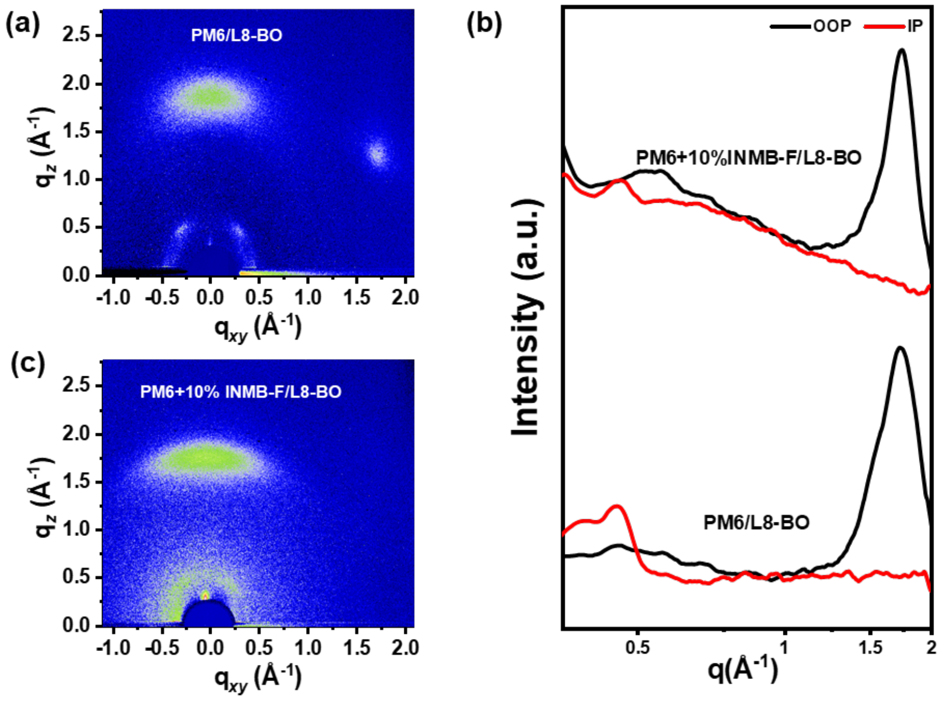

Supplementary Fig. 25. (a, c) 2D GIWAXS patterns and (b) 1D profiles of PM6/L8-BO films.

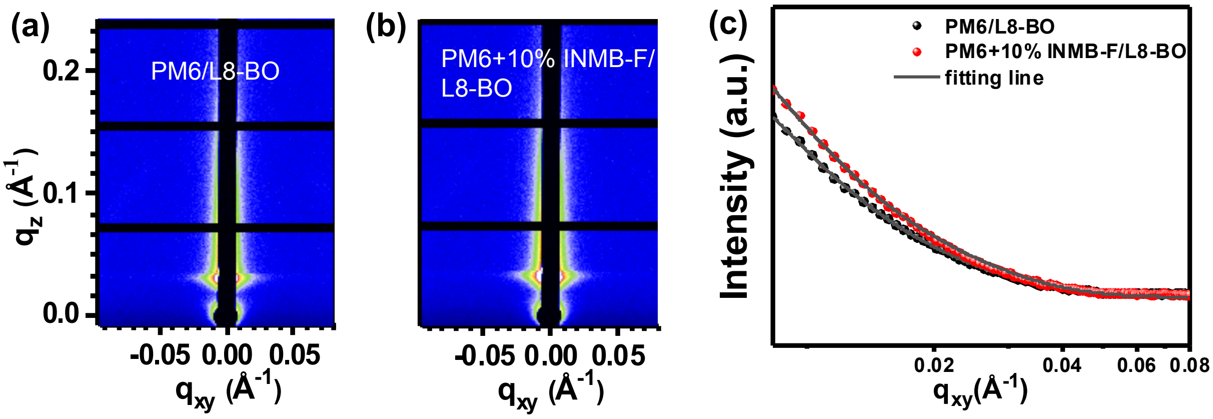

Supplementary Fig. 26. (a,b) 2D GISAXS patterns and (c)1D profiles of PM6/L8-BO films.

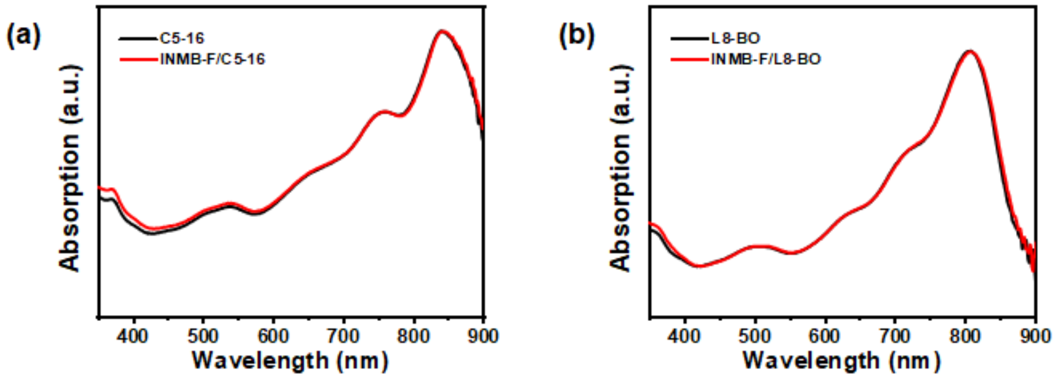

Supplementary Fig. 27. Absorption spectra of (a) INMB-F/C5-16 and (b) INMB-F/L8-BO films by spin-coating INMB-F on substrate that is followed by the casting of NFA on INMB-F.

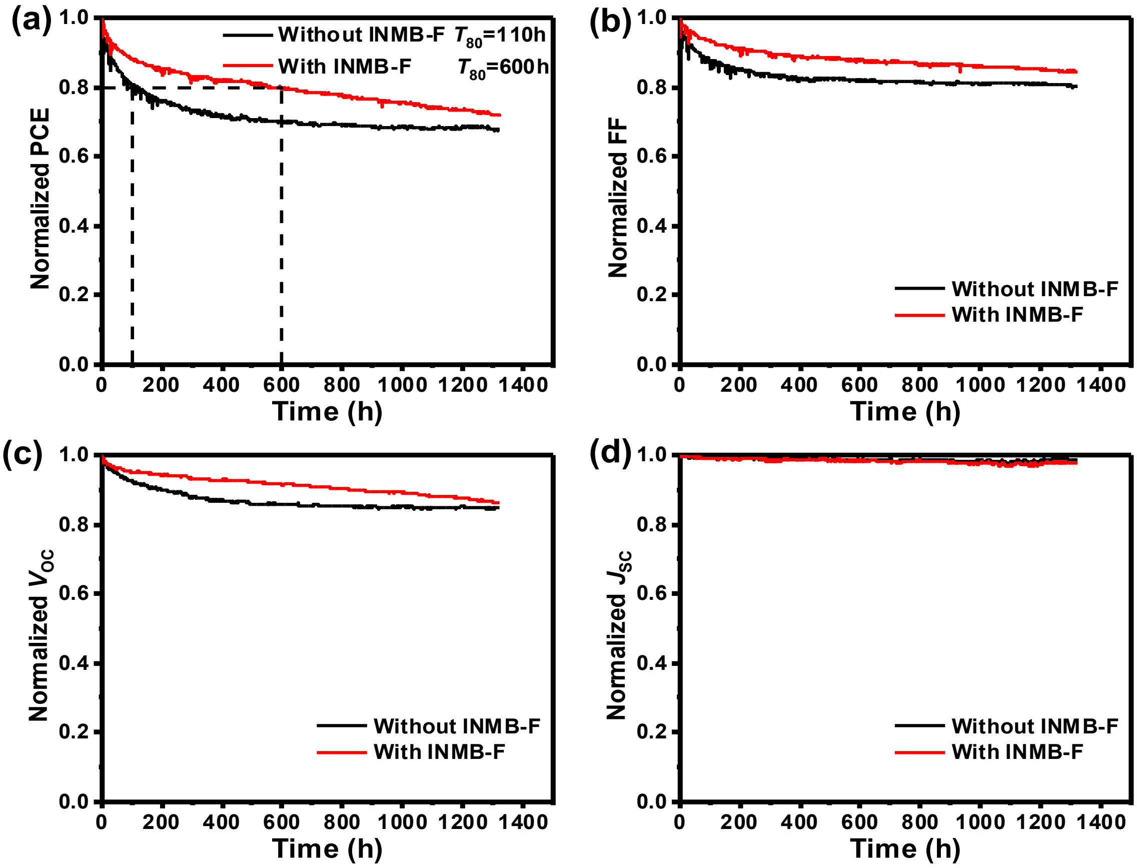

Supplementary Fig. 28. Normalized (a) PCE, (b) FF, (c) V_{OC}, and (d) J_{SC} of the PM6/L8-BO device processed without/with INMB-F for continuous light illumination over 1300 h.

Supplementary Tables

Supplementary Table 1. Summary of contact angles (θ), and surface tensions (γ) of C5-16, L8-BO, PM6, D18-Cl, PTB7-Th, and INMB-F neat films, and compatibility (χ) of C5-16, L8-BO, PM6, D18-Cl, PTB7-Th with INMB-F.

Materials	Contact Angle (°) (Water)	Contact Angle (°) (EG)	γ (mN/m)	χ
C5-16	96.3	70.0	25.8	
L8-BO	95.1	69.5	25.3	
PM6	105.7	77.4	26.6	
D18-Cl	104.6	76.7	25.5	
PTB7-Th	98.6	71.6	26.1	
INMB-F	66.7	28.3	43.1	
C5-16+INMB-F	-	-	-	2.2
L8-BO+INMB-F	-	-	-	2.3
PM6+INMB-F	-	-	-	2.0
D18-Cl+INMB-F	-	-	-	2.3
PTB7-Th+INMB-F	-	-	-	2.1

Supplementary Table 2. Hole mobilities of PM6, D18-Cl and PTB7-Th with/without INMB-F.

Active layer	μ_h (10^{-4} cm^{-1}V^{-1}s^{-1})	μ_e (10^{-4} cm^{-1}V^{-1}s^{-1})
PM6	5.6	/
PM6+INMB-F	8.2	/
D18-Cl	5.3	/
D18-Cl+INMB-F	6.6	/
PTB7-Th	4.6	/
PTB7-Th+INMB-F	6.0	/

Supplementary Table 3. Photovoltaic performance of PM6/C5-16 devices with different amount of INMB-F.

System	PCE (%)	FF (%)	J_{SC} (mA cm^{-2})	$J_{SC}^{cal.}$ (mA cm^{-2})	V_{OC} (V)
PM6/C5-16	17.8(17.6±0.2)	78.5(77.8±0.8)	27.18(26.99±0.17)	26.21	0.835(0.834±0.002)
PM6+10% INMB-F/C5-16	18.5(18.2±0.3)	80.2(79.5±0.6)	27.65(27.45±0.17)	26.58	0.834(0.833±0.001)
PM6+20% INMB-F/C5-16	13.5(13.2±0.3)	69.1(68.7±0.4)	24.26(23.59±0.61)	23.13	0.807(0.805±0.002)

Note: Average values with standard deviation were obtained from 16 individual devices.

Supplementary Table 4. Photovoltaic performance of PM6/C5-16 devices with and without INMB-F upon different TA temperatures.

System	treatment	PCE (%)	FF (%)	J_{sc} (mA cm^{-2})	J_{SC}^{cal} (mA cm^{-2})	V_{oc} (V)
PM6/C516	As cast	17.6(17.5±0.1)	78.0(77.5±0.5)	26.89(26.72±0.14)	25.85	0.842(0.84±0.003)
	80 °C TA	17.8(17.6±0.2)	78.5(77.8±0.8)	27.18(26.99±0.17)	26.21	0.835(0.834±0.002)
	100 °C TA	17.5(17.1±0.3)	78.3(77.8±0.5)	26.9(26.61±0.28)	25.61	0.830(0.827±0.002)
	120 °C TA	17.1(16.9±0.2)	77.9(77.5±0.5)	26.69(26.34±0.22)	25.73	0.822(0.819±0.002)
	150 °C TA	16.3(16.2±0.1)	77.6(77.4±0.2)	26.23(26.01±0.2)	25.08	0.806(0.803±0.003)
PM6+10% INMB-F/C516	As cast	18.2(18.1±0.1)	79.3(79.1±0.2)	27.23(27.11±0.11)	26.14	0.843(0.841±0.002)
	80 °C TA	18.5(18.2±0.3)	80.2(79.5±0.6)	27.65(27.45±0.17)	26.58	0.834(0.833±0.001)
	100 °C TA	17.8(17.6±0.2)	79.1(78.9±0.2)	26.87(26.52±0.26)	25.76	0.831(0.829±0.003)
	120 °C TA	17.2(17±0.2)	78.2(78.1±0.2)	26.78(26.47±0.33)	25.67	0.822(0.819±0.002)
	150 °C TA	16.4(16.2±0.2)	78.3(78±0.4)	26.0(25.81±0.19)	24.94	0.805(0.803±0.002)

Note: Average values with standard deviation were obtained from 16 individual devices.

Supplementary Table 5. Summary of recently reported high-performance PM6/L8-BO binary OSCs.

**Devices**	**PCE (%)**	**FF (%)**	**J_{SC} (mA cm^{-2})**	**V_{OC} (V)**	**Reference**
PM6:L8-BO	18.32	81.5	25.72	0.87	[2]
PM6:L8-BO	18.60	80.0	26.03	0.893	[3]
PM6:L8-BO	18.77	79.94	26.37	0.89	[4]
PM6:L8-BO	18.74	80.6	26.11	0.89	[5]
PM6:L8-BO	18.5	80.3	26.32	0.877	[6]
PM6/L8-BO	18.86	80.39	26.61	0.883	[7]
PM6:L8-BO	18.85	79.32	26.73	0.889	[8]
PM6:L8-BO	18.82	79.65	26.64	0.886	[9]
PM6:L8-BO	19.0	80.6	26.2	0.90	[10]
PM6:L8-BO	18.69	80.1	25.81	0.904	[11]
PM6:L8-BO	18.31	79.2	26.27	0.880	[12]
PM6:L8-BO	18.39	77.51	26.54	0.899	[13]
PM6/L8-BO	19.02	80.5	26.68	0.88	[14]
PM6:L8-BO	18.42	80.2	25.81	0.89	[15]
PM6:L8-BO	18.36	79.68	25.62	0.899	[16]
**PM6/L8-BO**	**19.4**	**81.3**	**26.94**	**0.883**	**This work**

Supplementary Table 6. Hole mobilities and electron mobilities of four system OSCs.

**Active layer**	μ_h (10^{-4} cm^{-1}V^{-1}s^{-1})	μ_e (10^{-4} cm^{-1}V^{-1}s^{-1})	μ_e/μ_h
PM6/C5-16	3.52	4.28	1.22
PM6+INMB-F/C5-16	4.10	4.61	1.12
PM6/L8-BO	4.23	5.30	1.25
PM6+INMB-F/L8-BO	5.03	5.61	1.11
D18-Cl/L8-BO	3.41	4.01	1.18
D18-Cl+INMB-F/L8-BO	4.03	4.33	1.07
PTB7-Th/C5-16	3.23	3.50	1.08
PTB7-Th+INMB-F/C5-16	3.75	3.82	1.02

Supplementary Table 7. Exciton dissociation (P_{diss}) and charge collection (P_{coll}) efficiencies of OSCs.

**System**	P_{diss} (%)	P_{coll} (%)
PM6/C5-16	98.9	84.7
PM6+INMB-F/C5-16	99.1	87.1
PM6/L8-BO	98.9	90.3
PM6+INMB-F/L8-BO	99.3	94.2
D18-Cl/L8-BO	98.6	84.4
D18-Cl+INMB-F/L8-BO	98.7	90.4
PTB7-Th/C5-16	98.3	80.3
PTB7-Th+INMB-F/C5-16	98.4	85.3

Supplementary Table 8. Fitting parameters of 1D GISAXS profiles of PM6/L8-BO films with/without INMB-F, where the correlation length ξ refers to the domain size of the PM6-rich phase, η and D represent the correlation length and fractal dimension of acceptors, and 2Rg is regarded as the domain size of acceptor domain [5].

	ξ (nm)	η (nm)	D	2R_g (nm)
PM6/L8-BO	15.6	2.8	3	13.7
PM6+10% INMB-F/L8-BO	18.3	4.1	3	20.6

Supplementary References

1. Cai, J. et al. Fluorinated solid additives enable high efficiency non-fullerene organic solar cells. *J. Mater. Chem. A* **8**, 4230-4238 (2020).
2. Li, C. et al. Non-fullerene acceptors with branched side chains and improved molecular packing to exceed 18% efficiency in organic solar cells. *Nat. Energy* **6**, 605–613 (2021).
3. Song, J. et al. High-efficiency organic solar cells with low voltage loss induced by solvent additive strategy. *Matter* **7**. 2542-2552 (2021).
4. Meng, H. et al. 18.77 % efficiency organic solar cells promoted by aqueous solution processed cobalt(ii) acetate hole transporting layer. *Angew. Chem. Int. Ed.* **60**, 22554 (2021).
5. Xu, X. et al. Polymer solar cells with 18.74% efficiency: from bulk heterojunction to interdigitated bulk heterojunction. *Adv. Funct. Mater.* **32**, 2108797 (2021).
6. He, C. et al. Manipulating the D:A interfacial energetics and intermolecular packing for 19.2% efficiency organic photovoltaics. *Energy Environ. Sci.* **15**, 2537-2544 (2022).
7. He, C. et al. Versatile sequential casting processing for highly efficient and sSupplementary Table binary organic photovoltaics. *Adv. Mater.* **34**, 2203379 (2022).
8. Li, C. et al. Achieving record-efficiency organic solar cells upon tuning the conformation of solid additives. *J. Am. Chem. Soc.* **144**, 14731-14739 (2022).
9. Zhang, G.et al. Co-La-based hole-transporting layers for binary organic solar cells with 18.82 % efficiency. *Angew. Chem. Int. Ed.* **62**, e202216304 (2023).
10. Song, X. et al. Solvent-induced anti-aggregation evolution on small molecule electron - transporting layer for efficient, scalable, and robust organic solar cells. *Adv. Energy Mater.* **13**, 2203009 (2022).
11. Xu, X. et al. Sequential deposition of multicomponent bulk heterojunctions increases efficiency of organic solar cells. *Adv. Mater.* **35**, 2208997 (2023).
12. Ma, R. et al. Revealing the underlying solvent effect on film morphology in high-efficiency organic solar cells by combined ex-situ and in-situ observations. *Energy Environ. Sci.* **16**, 2316-2326 (2023).
13. Pang, B. et al. Benzo[d]thiazole based wide bandgap donor polymers enable 19.54% efficiency organic solar cells along with desirable batch-to-batch reproducibility and general applicability. *Adv. Mater.* **35**, 202300631 (2023).

14. Ding, G. et al. Solid additive-assisted layer-by-layer processing for 19% efficiency binary organic solar cells. *Nano-Micro Lett.* **15**, 92 (2023).
15. Wang, Y. et al. Easy isomerization strategy for additives enables high-efficiency organic solar cells. *Adv. Energy Mater.* **13**, 2300524 (2023).
16. Yang, L. et al. Doped/undoped A1-A2 typed copolymers as ETLs for highly efficient organic solar cells. *Adv. Funct. Mater.*, 2303603 (2023).
