# Supplementary material for: Electrostatic force promoted intermolecular stacking of polymer donors toward 19.4% efficiency binary organic solar cells
Source: Nat Commun. 2023 Oct 9;14:6297. doi: 10.1038/s41467-023-42071-2 (PMC10562425; doi:10.1038/s41467-023-42071-2)
Supplement: Supplementary file 3 — Solar Cells Reporting Summary [file 41467_2023_42071_MOESM3_ESM.pdf]

nature research

Corresponding author(s): Tao Wang

Solar Cells Reporting Summary

Nature Research wishes to improve the reproducibility of the work that we publish. This form is intended for publication with all accepted papers reporting the characterization of photovoltaic devices and provides structure for consistency and transparency in reporting. Some list items might not apply to an individual manuscript, but all fields must be completed for clarity.

For further information on Nature Research policies, including our data availability policy, see Authors & Referees.

Experimental design

Please check: are the following details reported in the manuscript?

1. Dimensions

Area of the tested solar cells
☒ Yes — The active layer size is 6.625mm2 and an aperture mask with the illumination area of 4.04 mm2
☐ No — *Explain why this information is not reported/not relevant.*

Method used to determine the device area
☒ Yes — The area of our mask was determined at the National Photovoltaic Product Quality Inspection & Testing Center (China)
☐ No — *Explain why this information is not reported/not relevant.*

2. Current-voltage characterization

Current density-voltage (J-V) plots in both forward and backward direction
☐ Yes — Only the plots in forward direction was supplied.
☒ No — OPVs do not usually have hysteresis.

Voltage scan conditions
For instance: scan direction, speed, dwell times
☒ Yes — See details in Section "Instruments and measurements".
☐ No — *Explain why this information is not reported/not relevant.*

Test environment
For instance: characterization temperature, in air or in glove box
☒ Yes — Our devices were characterized at room temperature (ca. 25 Celsius degree) in air.
☐ No — *Explain why this information is not reported/not relevant.*

Protocol for preconditioning of the device before its characterization
☒ Yes — Solar simulator was stabilised for 30 mins before J-V testing.
☐ No — *Explain why this information is not reported/not relevant.*

Stability of the J-V characteristic
Verified with time evolution of the maximum power point or with the photocurrent at maximum power point; see ref. 7 for details.
☒ Yes — We tested the operational stability.
☐ No — *Explain why this information is not reported/not relevant.*

3. Hysteresis or any other unusual behaviour

Description of the unusual behaviour observed during the characterization
☐ Yes — *State where this information can be found in the text.*
☒ No — No hysteresis or other unusual behaviour was observed during the characterization of our OPVs.

Related experimental data
☒ Yes — Any related experimental data is available from authors upon requests.
☐ No — *Explain why this information is not reported/not relevant.*

4. Efficiency

External quantum efficiency (EQE) or incident photons to current efficiency (IPCE)
☒ Yes — As shown in Fig. 4e-h, Supplementary Fig. 12
☐ No — *Explain why this information is not reported/not relevant.*

A comparison between the integrated response under the standard reference spectrum and the response measure under the simulator
☒ Yes — As shown in Table 1 and Supplementary Table 3 and Supplementary Table 4
☐ No — *Explain why this information is not reported/not relevant.*

For tandem solar cells, the bias illumination and bias voltage used for each subcell
☐ Yes — *State where this information can be found in the text.*
☒ No — We only fabricated single-junction solar cells.

5. Calibration

Light source and reference cell or sensor used for the characterization	☒ Yes	See details in Section "Instruments and measurements".
	☐ No	*Explain why this information is not reported/not relevant.*
Confirmation that the reference cell was calibrated and certified	☒ Yes	See details in Section "Instruments and measurements".
	☐ No	*Explain why this information is not reported/not relevant.*
Calculation of spectral mismatch between the reference cell and the devices under test	☐ Yes	*State where this information can be found in the text.*
	☒ No	The light spectrum used for measurements matches well with the reference silicon cell, and we did not calculate the spectral mismatch between the reference cell and the tested devices.

6. Mask/aperture

Size of the mask/aperture used during testing	☒ Yes	We used a mask with 4 mm2 for measuring photovoltaic performance.
	☐ No	*Explain why this information is not reported/not relevant.*
Variation of the measured short-circuit current density with the mask/aperture area	☐ Yes	*State where this information can be found in the text.*
	☒ No	We measured all devices with the same mask.

7. Performance certification

Identity of the independent certification laboratory that confirmed the photovoltaic performance	☒ Yes	We conducted the performance certification.
	☐ No	*Explain why this information is not reported/not relevant.*
A copy of any certificate(s)<br>*Provide in Supplementary Information*	☒ Yes	See Supplementary Fig. 14
	☐ No	*Explain why this information is not reported/not relevant.*

8. Statistics

Number of solar cells tested	☒ Yes	Number of solar cells tested is provided in Table 1.
	☐ No	*Explain why this information is not reported/not relevant.*
Statistical analysis of the device performance	☒ Yes	Statistical results of the devices are listed in Table 1.
	☐ No	*Explain why this information is not reported/not relevant.*

9. Long-term stability analysis

Type of analysis, bias conditions and environmental conditions<br>*For instance: illumination type, temperature, atmosphere humidity, encapsulation method, preconditioning temperature*	☒ Yes	We tested the operational stability.
	☐ No	*Explain why this information is not reported/not relevant.*
